# A new 3D method for measuring cranio-facial relationships with cone beam computed tomography (CBCT)

**DOI:** 10.4317/medoral.18671

**Published:** 2013-03-25

**Authors:** Natalia Zamora, Rosa Cibrián, Jose L. Gandia, Vanessa Paredes

**Affiliations:** 1 Orthodontist , Research Assistant Professor, Department of Orthodontics, Faculty of Medicine and Dentistry, University of Valencia, Valencia, Spain; 2 Associate Professor, Department of Physiology, Faculty of Medicine and Dentistry, University Valencia, Valencia, Spain; 3 Orthodontist, Professor and Department Chair of Postgraduate Orthodontics Masters Course, Department of Orthodontics, Faculty of Medicine and Dentistry, University of Valencia, Valencia, Spain; 4 Orthodontist and PhD Associate Professor, Department of Orthodontics, Faculty of Medicine and Dentistry, University of Valencia, Valencia, Spain

## Abstract

Objectives: CBCT systems, with their high precision 3D reconstructions, 1:1 images and accuracy in locating cephalometric landmarks, allows us to evaluate measurements from craniofacial structures, so enabling us to replace the anthropometric methods or bidimensional methods used until now. The aims are to analyse cranio-facial relationships in a sample of patients who had previously undergone a CBCT and create a new 3D cephalometric method for assessing and measuring patients. Study Design: 90 patients who had a CBCT (i-Cat®) as a diagnostic register were selected. 12 cephalometric landmarks on the three spatial planes (X,Y,Z) were defined and 21 linear measurements were established. Using these measurements, 7 triangles were described and analysed. With the sides of the triangles: (CdR-Me-CdL); (FzR-Me-FzL); (GoR-N-GoL); and the Gl-Me distance, the ratios between them were analysed. In addition, 4 triangles in the mandible were measured (body: GoR-DB-Me and GoL-DB-Me and ramus: KrR-CdR-GoR and KrL-CdL-GoL). Results: When analyzing the sides of the CdR-Me-CdL triangle, it was found that the 69.33% of the patients could be considered symmetric. Regarding the ratios between the sides of the following triangles: CdR-Me-CdL, FzR-Me-FzL, GoR-N-GoL and the Gl-Me distance, it was found that almost all ratios were close to 1:1 except between the CdR-CdL side with respect the rest of the sides. With regard to the ratios of the 4 triangles of the mandible, it was found that the most symmetrical relationships were those corresponding to the sides of the body of the mandible and the most asymmetrical ones were those corresponding to the base of such triangles. Conclusions: A new method for assessing cranio-facial relationshps using CBCT has been established. It could be used for diverse purposes including diagnosis and treatment planning.

** Key words:**Craniofacial relationship, CBCT, 3D cephalometry.

## Introduction

Facial symmetry, is one element of bodily symmetry, including fluctuating asymmetry. Along with traits such as averageness and youthfulness it influences judgments of aesthetic traits of physical attractiveness and beauty, and is associated with fitness-linked traits including health(1). It is also hypothesized as a factor in both interpersonal attraction and interpersonal chemistry.

British orthodontist R.J. Edler (2) cited research supporting the claim that bilateral symmetry is an important indicator of freedom from disease, and worthiness for mating. Random differences between the two sides, known in biological terms as fluctuating asymmetry, and not deliberate asymmetrical structures found in some animals, develops throughout the lifespan of the individual and is a sign of the phenotype being subjected to some levels of stress.

The ability to cope with these pressures is partly reflected in the levels of symmetry. “Perfect beauty” exists for each person when all the exact relationships of beauty are met. A higher degree of symmetry indicates a better coping system for environmental factors. While the visible signs of this may not be particularly apparent, it is thought that they have at least an unconscious effect on people’s perception of their beauty.

Ricketts (3) in 1982, described “the divine proportion” (analysis of the divine rectangle, pentagon and triangle) by undertaking a facial analysis based on both, facial width and height ratios in soft tissues. Likewise, he found those divine proportions on the skeletal level, both sagittal and frontal. On the sagittal level, he found eight divine proportions and on the frontal level three divine proportions. In this way, he found “the divine proportion” by locating various frontal and lateral measurements on the cranium and in the soft tissues of the human face.

Perrett et al.(4) showed that beauty consists of a more complex concept, as they discovered in a study that the average number of faces of all the women studied was less attractive than the average of the faces that separately were considered attractive.

Jefferson (5) revised divine proportions both in soft and hard tissues claiming that the normal face was the most attractive given that it was made up of “divine proportions” and this provided it with a more healthy, strong and fertile look.

Hönn and Göz (6) determined to what extent the beauty of a face could be measured and how symmetry provided a tool for doing so. In undertaking these measurements of facial proportions, they found that various authors stated that anthropometric methods were preferable to cephalometric measurements since they involved non-invasive methods and provided a three dimensional assessment of the structures under study.

Despite these claims of Hönn and Göz (6), with the appearance of Cone Beam Computed Tomography (CBCT) with its high precision (1:1) and accurately locating the cephalometric points bringing them over more to the 3D reality. (7-16) it is now possible to assess different measurements and facial relationships in patients.

There have been several studies that have attempte bringing them over more to the 3D reality to design cephalometric analysis in 3D, some of them adapting conventional 2D cephalometric analysis to 3D, using the visualization of craniofacial structures in three dimensions, while others have designed new measurements for the study of cleftpalate patients, patients with asymmetries or orthognatic surgical candidates, or as part of pilot studies for other aplications.

The development and implement of this 3D cephalometry will mean progression in daily practice since measurements will be done directly in three-dimensional reconstruction of the patient with the many advantages that this implies. It is a matter of fact thet a 3D structure always gives us a more realistic view from what we face.

The aims of this study were to describe and analyse the relationshps between different measurements correspongding to cranio-facial structures in a sample of patients who had undergone CBCT.

## Material and Methods

A study approved by the ethical committee of the Clinical University Hospital of the University of Valencia was undertaken. An initial sample consisted of 122 CBCTs of patients who had previously undergone a full cranial scan at the University of Valencia, Spain. The CBCTs used were obtained from the data base of those patients who had previously undergone such diagnostic tool because different reasons: included teeth like canines or third molars, agenesia or supernumerary teeth, all without moderate to severe skeletal asymmetries. No patient was scanned because of the purpose of the present study.

From this initial sample, 32 CBCTs were excluded for the following reasons.

9 Patients with open mouth.

11 Patients with multiple missing teeth.

12 Patients with moderate to severe asymmetries.

The final sample was composed of 90 CBCTs of patients between the ages of 8 and 50. The mean age was (18.05 years ± 8.69).

Each of the patients had undergone a scan using the i-CAT® (Imaging Sciences International, Hatfield, Pa) equipment. This CBCT device uses an amorphous silicone flat panel sensor to capture the fields of view (FOV). The field of view (FOV) employed was the portrait mode that captures data in extended FOV mode and includes the full head of 170 mm in height x 230 mm in diameter with a scanning time of 8.9 seconds. It was set at medium quality and high resolution mode. It generates a total of 326 slices, with an image matrix size of 400x400. The voxel size is of 0.4 x 0.4 x 0.4 mm. The focal size is 0.5mm y and the size of its base is 119x142 cm. Tube voltage is 120 kV and its intensity 23.87 mAs. The size of the data files generated is in the order of 35 megabytes.

The raw data and the slices obtained from the CBCTs were imported to the InVivo5® software (Anatomage, San Jose, CA) which was used to visualize the slices and three dimensional images that are obtained from a CBCT. This is where the three-dimensional reconstruction of the DICOM images (Digital Imaging and Communications in Medicine) is made.

In order to undertake this particular study, 12 cephalometric points were defined on each of the three spatial planes (X, Y, Z) ( Table 1). The procedure for locating each point requires the selection of the most appropriate view or plane (sagittal, coronal, axial) and then adjusting that point on the other planes for better accuracy. Employing this location method, all the spatial positions of each point have been pinpointed on each of these axes as numerical values.

**Table 1:**
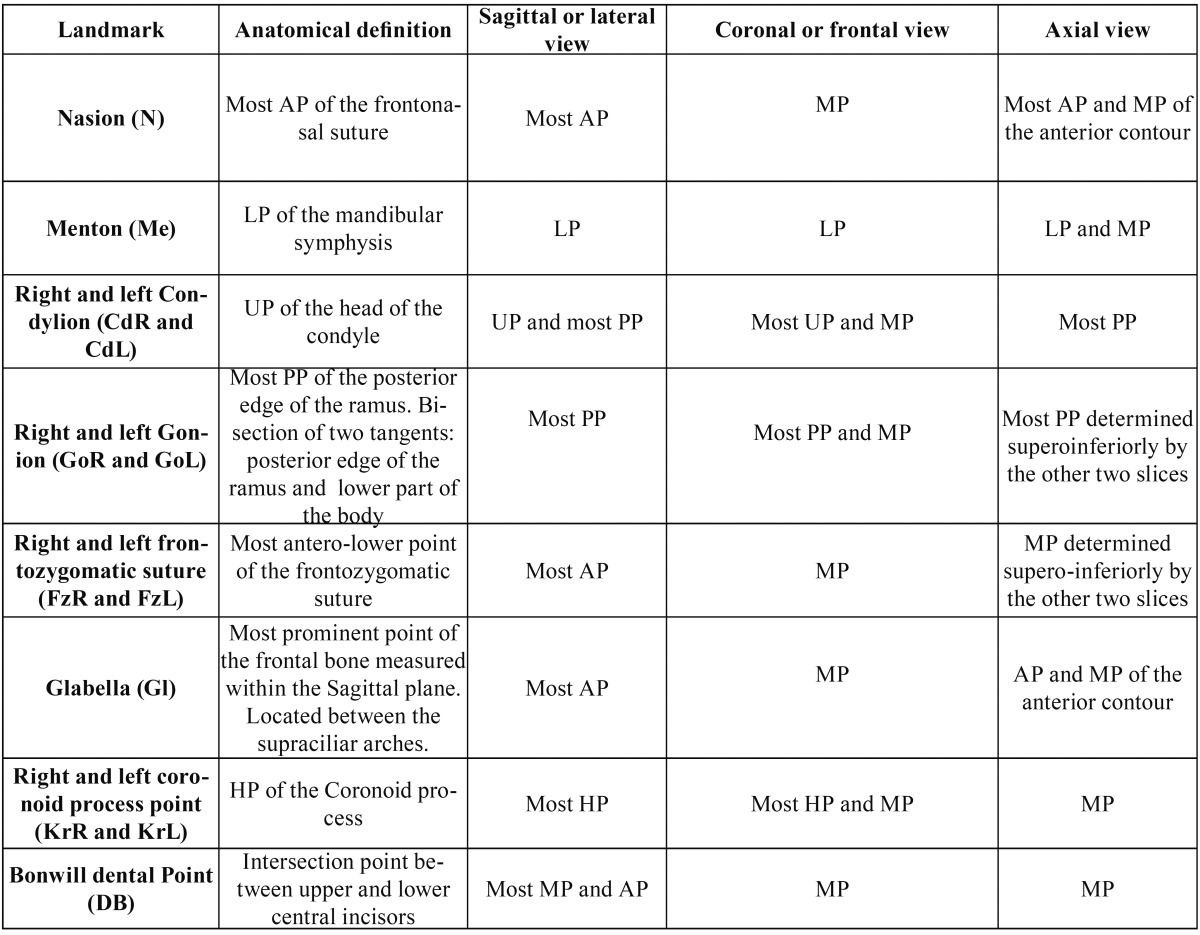
Definition of the three spatial planes of the 12 points used in this study. Anterior point (AP), midpoint (MP), posterior point (PP), lowest point (LP), upper point (UP), highest point (HP).

In a previous study undertaken by the same authors (17), the reproducibility and reliability in locating cephalometric points was analyzed. To assess the reproducibility on landmark location and the differences in the measurements, two observers with the same background and six years of experience in the field of orthodontics located 41 landmarks at three separate times. A total of 11,070 data were processed using the 15.0 SPSS statistical package®. To discover the reproducibility of the method on landmark location, an ANOVA analysis was undertaken using two factors of variation: time (t1, t2 and t3) and observers (Ob1 and Ob2) for each axis (X, Y and Z) and landmark. The order of the CBCT scans submitted to the observers (Ob1, Ob2) at t1, t2, t3 were different and randomly allocated. The intraclass correlation coefficient (ICC) was calculated. The results of the study showed that both intra-and inter-observer reliability were high, both being ICC ? 0.99. It was also found that the best ICC frequency was on axis Z.

Once the cephalometric points have been adapted to the 3D reality, that is, have been defined on each of the spatial planes (X, Y, Z) and have been duly located in their correct position, the next step to design a 3D cephalometric analysis of the craniofacial relationships, was to define 21 linear measurements ( Table 2).

**Table 2:**
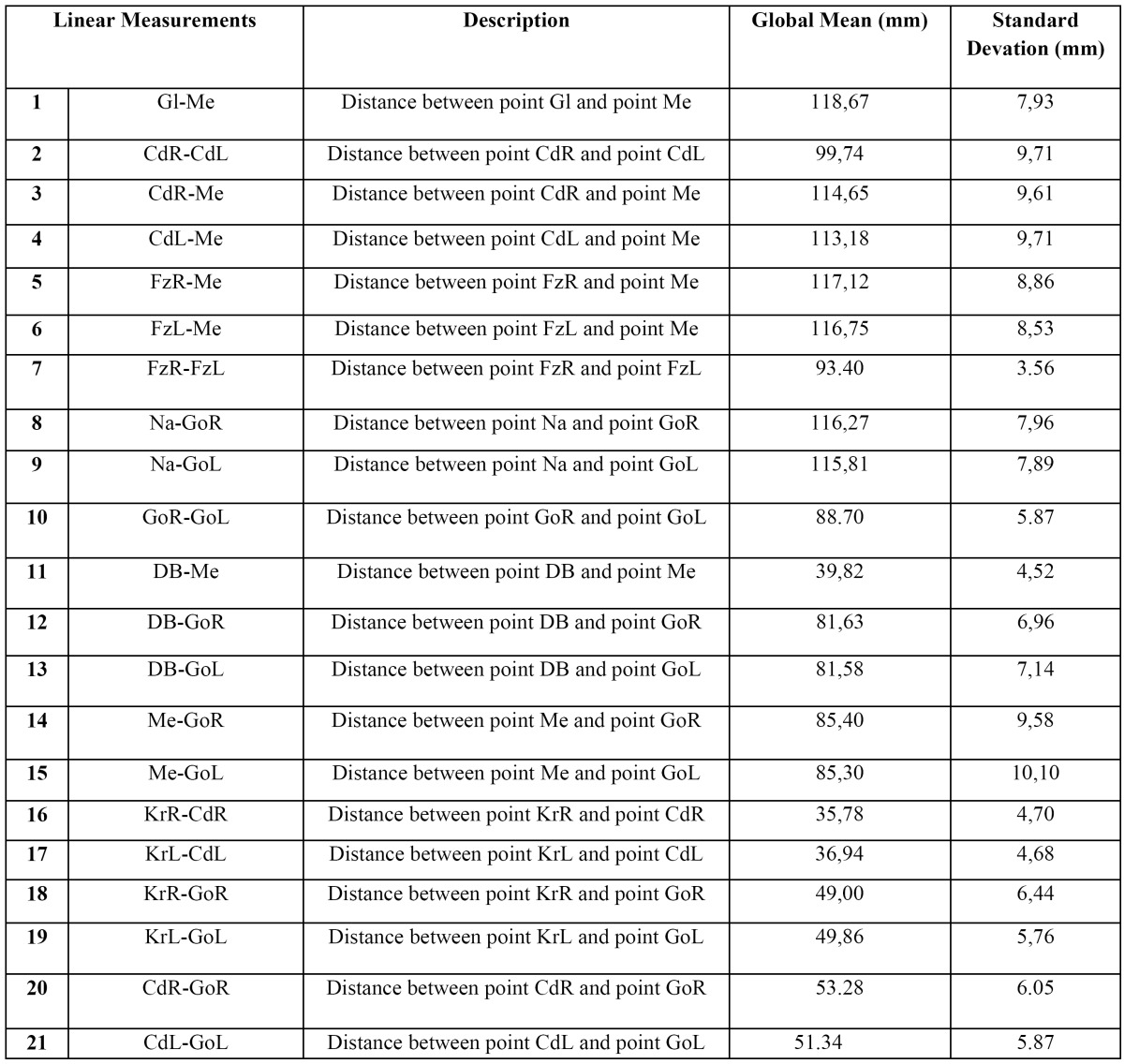
Description, global mean (mm) and standard deviation of the mean (mm) of the 21 linear measurements used in this study.

**Figure 1:**
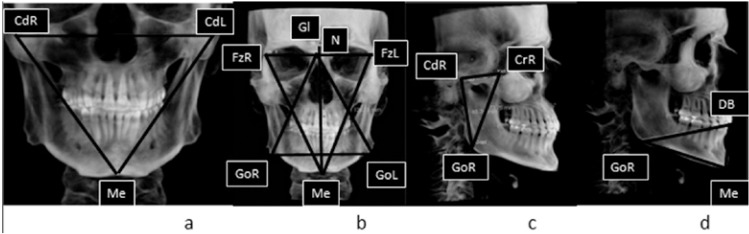
a) CdR-Me-CdL triangle, b) FzR-Me-FzL triangle, GoR-N-GoL triangle and Gl-Me distance c) Triangle of the mandibular ramus: CdR-KrR-GoR d) Triangles of the mandibular body: DB-Me-GoR; DB-Me-GoL.

After that, using those 21 linear measurements 7 triangles were created (Fig. 1).

1 Triangle formed by the CoR-Me-CoL points (Fig. 1).

1 Triangle formed by the FzR- Me-FzL points (Fig. 1).

1 Triangle formed by the GoR- N-GoL points (Fig. 1).

2 Triangles of the mandibular ramus: formed by the KrR-CoR-GoR points on the right side and the KrL-CoL-GoL points on the left side (Fig. 1).

2 Triangles of the mandibular body: formed by the DB-Me-GoR points on the right side and the DB-Me-GoL points on the left side (Fig. 1).

An Excel ® sheet, version 12.0 for Windows (Microsoft Corporation), was created to introduce all the variables and measurements obtained in the 3D cephalometric analysis of the 90 patients After that all data were introduced in version 17.0 of the statistical package SPSS ® for Windows (SPSS Inc., Chicago, IL). The means and standard deviations for each of the measurements were obtained. Finally, the correlations between variables were found using the Bonferroni and Scheffe analyses.

## Results

Once the 3D analysis was created, all the values of the different linear measurements and triangles were analysed.  Table 2 shows the means and overall typical deviations for the 21 linear measurements used in this study.

To eliminate bias in the study, and to be able to compare between subjects of different ages we only analysed angles and ratios between distances.

Firstly, we studied the relationships between the sides of the CdR-Me-CdL triangle (Fig. 1); this triangle gives information about the shape and position of the mandible.

To consider the mandible symmetric, we have analyzed the value of the lower angle of the triangle, the CdR-Me-CdL angle.

By doing this, we were searching for the symmetry of the left and right sides of the mandible.

**Figure 2:**
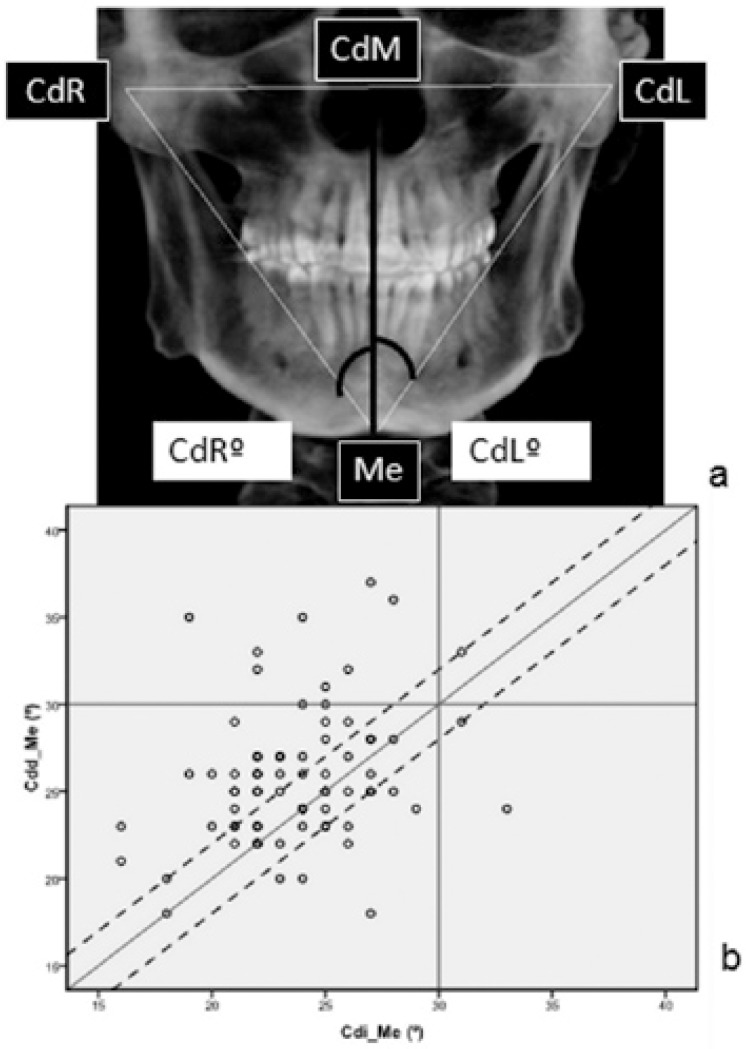
a) Distribution of semiangles (CdR-Me-CdM; CdL-Me-CdM) of the CdR-Me-CdL triangle. b) relationship between the semiangles. Diagonal line: represents perfect symmetry between sides. Dotted line: included those patients with ? 4º of discrepancy between right and left side.

When dividing this particular angle into two semiangles (Fig. 2), to be considered symmetrical, they should measure the same.

A midpoint in between the CdR-CdL line (CdM point) was determined. Then, a line starting on this point and finishing in Me was used to calculate the values of the two semiangles: CdR-Me-CdM and CdL-Me-CdM (Fig. 2).

2 shows the relationship between the semiangles. The diagonal line represents perfect symmetry between sides. Dotted lines included those patients with ? 4º of discrepancy between right and left side.

In our study, we observed that with this approach the 69.33% of the patients could be considered symmetric.

Secondly, we wanted to study if there was any type of relationship between the sides of the triangles formed by the CdR-Me-CdL; GoR-N-GoL; FzR-Me-FzL points and the Gl-Me distance. Some authors (3,6) in their studies and reviews stated that, in order to reach symmetry, a ratio of 1:1 between all sides must be achieved.

In our study, to measure this characteristic, the minimum, maximum and mean value of the different ratios between sides was assessed ( Table 3).

**Table 3:**
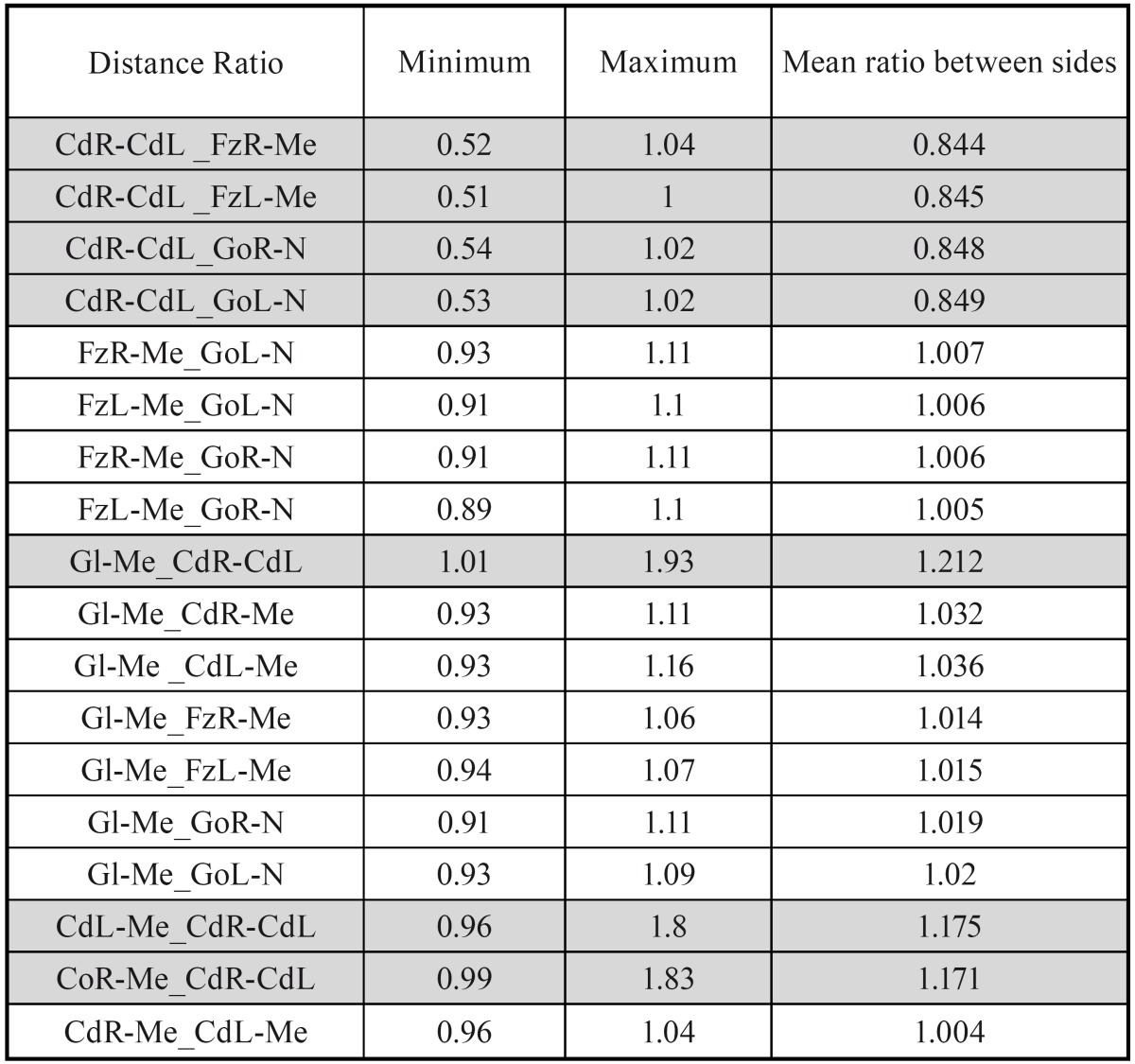
Minimum, maximum and mean value of the ratios between distances. The most asymmetrical ratios are highlighted in grey.

We observed that, in our sample, all the ratios studied between the different sides that form these triangles, presented values close to 1:1, indicating, thus, that those distances were similar to each other. Only the CdR-CdL distance did not approach a 1:1 ratio when compared with the values of the other distances.

We have also compared the mean difference (in mm) between right and left sides of the following distances: FzR-Me; FzL-Me; GoR-N; GoL-N; CdR-Me y CdL-Me. According to this, 66.66% of the cases studied present a maximun discrepancy of 5mm between right and left sides.

Thirdly, we analysed the ratios between the ramus and body triangles of the mandible (KrR-CdR-GoR and KrL-CdL-GoL) and (DB-Me-GoR and DB-Me-GoL). (Fig. 1).

A total of 9 relationships between these last four triangles were recorded.  Table 4 shows the values of all ratios. In dark grey the most symmetrical relationships have been represented. Those corresponding to the sides of the body of the mandible (DB-GoR with DB-GoL and Me-GoR with Me-GoL) are considered the most symmetrical ones. In light grey, the most asymmetrical relationships, those corresponding to the bases of such triangles, are represented (relationship between KrL-CdL and DB-Me and relationship between KrR-CdR and DB-Me).

**Table 4:**
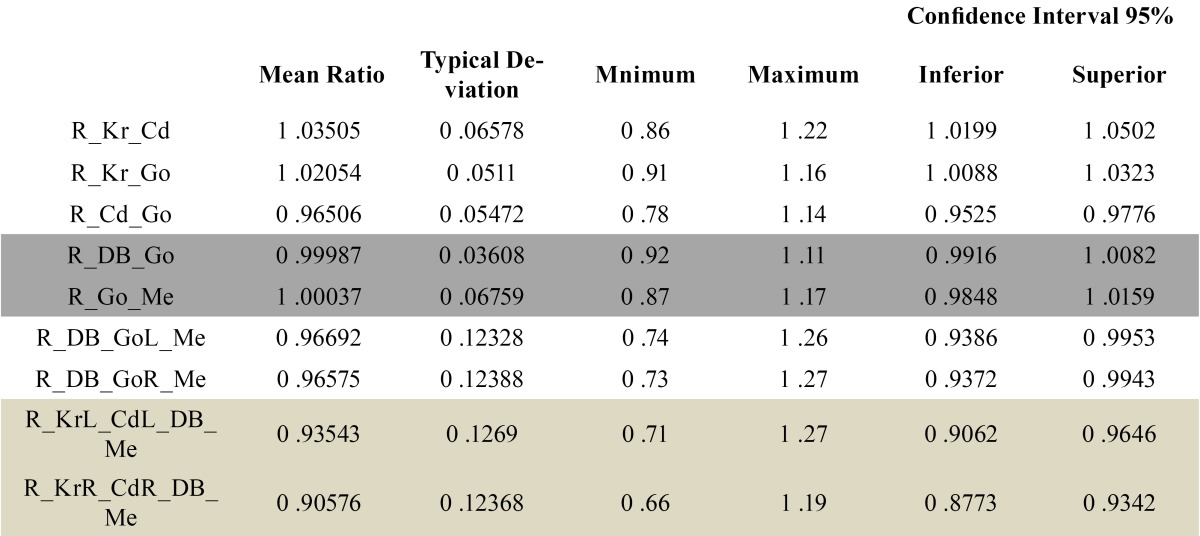
Analysis of the 9 relationships of all sides that form the four triangles of the body and ramus of the mandible.. R_Kr_Cd= relationship between KrL-CdL and KrR-CdR; R_Kr_Go= relationship between KrL-GoL and KrR-GoR; R_Cd_Go= relationship between CdL-GoL and CdR-GoR; R_DB_Go= relationship between DB-GoL and DB-GoR; R_Go_Me= relationship between GoL-Me and GoR-Me; R_DB_GoL_Me= relationship between DB-GoL and GoL-Me; R_DB_GoR_Me= relationship between DB-GoR and GoR-Me; R_KrL_CdL_DB_Me= relationship between KrL-CdL and DB-Me; R_KrR_CdR_DB_Me= relationship between KrR-CdR and DB-Me.

## Discussion

To carry out this study, records of patients who had previously undergone a CBCT as a diagnostic tool because some additional alteration (agenesia, supernumerary teeth, included teeth…) were used; so undertaking a CBCT was justified. Patients with moderate and severe asymmetries were not included in this study. Despite the fact that the sample was large, including 90 patients, the drawback of carrying out a study of these characteristics is that irradiating actual patients only for research purposes is not justified.

In the present study, only angles and ratios have been assed. Consequently, no age groups have been made as there is no difference between ages when measuring ratios and angles (3.4.6).

Acording to some authors who studied the symmetry of the skull, (3,4,6) it was found that in skulls with perfect symmetry the mandible must respond to geometric triangulation; in our study we examinated the mandible as a triangle (CdR-Me-CdL) of an equilateral type whose sides must be equal.

However, it is difficult for people to have such perfect symmetry, because of multiple influencing factors (1-2,4,6). We considered that measuring the angles of the mandible is a good method of comparing sides and symmetry. It is clear that, the more equal the angles between sides are, the more symmetry we will find in a mandible. With respect to the first triangle analysed (CdR-Me-CdL), we have found that the major part of our sample can be considered symmetric. Particularly, having the two semiangles of the lower angle (CdR-Me-CdM/ CdL-Me-CdM) equal or almost equal means that the other two upper angles that form that triangle must be equal as well, and this means symmetry.

Secondly, on analysing the ratios existing between each of the sides of the following triangles: CdR-Me-CdL; FzR-Me-FzL; GoR-N-GoL and the Gl-Me distance, in order to reach symmetry, each of the sides must measure the same. We found, in our study, that almost all ratios between distances were close to 1:1. Only the relationship between the CdR-CdL distance with respect the rest of the distances, wasn´t close to 1:1.

If we interpret these values, we can find that, in the majority of the patients, a good relationship between different structures is present, which as some authors stated, means symmetry and aesthetics (3,5,6). This could be a good method, thus, to evaluate the characteristics of a particular patient, when looking for a complete diagnosis.

Finally, we have analysed the four isosceles triangles obtained from the mandibular body and ramus and the relationships that existed between sides. In this study, all the ratios existing between the sides that form these 4 triangles of the body and ramus of the mandible have been established.

On analysing the ratios between the base of the mandibular body and ramus, it was found that the most symmetrical relationships were those corresponding to the sides of the body of the mandible and the most asymmetrical relationships were those corresponding to the base of such triangles.

With the introduction of CBCT in orthodontic diagnosis, new possibilities have opened up in the study of cranio-facial relationships (8,13). We considered it of interest to create and study a sample of patients using these new technologies. This type of 3D analysis are going to be implemented in the future, for all the patients who need a CBCT. It is, thus, important to create norms and measurements in order to diagnose them following also these criteria.

The conclusions of our study are.

We observed that regarding to the CdR-Me-CdL triangle, the 69.33% of the patients could be considered symmetric.

As regards the relationship between the sides of the CdR-Me-CdL, FzR-Me-FzL, GoR-N-GoL triangles and the Gl-Me distance,it was found that almost all ratios were close to 1:1. Only the relationship between the CdR-CdL distance with respect the rest of the distances, wasn't close to 1:1.

With regard to the ratios of the 4 triangles of the mandible, it was found that the most symmetrical relationships were those corresponding to the sides of the body of the mandible and the most asymmetrical relationships were those corresponding to the base of such triangles.

To conclude, it must be said that we did not found articles regarding the topic of the present study. Only few authors had studied the same topic in the past, but using measurements directly made on skulls, or using cephalometry with 2D technologies (3-6), which means that comparisons of our results could not be made as extensevely as we would like.
